# Harmonic-Selective Gaussian Filtering for Morphology and Timing Preservation in PPG Signals

**DOI:** 10.3390/s26123710

**Published:** 2026-06-11

**Authors:** Sarai Dominguez-Hernandez, Gonzalo Paez, Moises Padilla

**Affiliations:** Centro de Investigaciones en Optica, A.C., Loma del Bosque 115, Lomas del Campestre, Leon C.P. 37150, Guanajuato, Mexico

**Keywords:** photoplethysmography, harmonic components, waveform morphology, signal timing, zero-phase filter, PPG, frequency domain, Gaussian filter, dicrotic notch, systole, diastole

## Abstract

Photoplethysmography (PPG) is a widely used non-invasive optical technique for assessing cardiovascular dynamics and related hemodynamic processes. However, conventional noise-reduction methods can alter signal timing and distort waveform morphology, thereby affecting the identification of physiologically relevant events. Here, we propose a frequency-domain Gaussian filtering framework for selectively extracting harmonics from PPG signals. The method combines a Gaussian band-reject filter centered at 0 Hz to suppress the dominant DC component, reduce the non-pulsatile baseline, and attenuate low-frequency contributions associated with slow modulation processes. Symmetric Gaussian bandpass filters are then applied to isolate harmonic components, with the number of retained bands adapted to the requirements of a given application. As a proof of concept, the framework was applied to both a simulated PPG waveform and an experimental PPG recording. Because of the symmetric zero-phase properties of the filters, the temporal alignment of key PPG events, including the systolic peak, diastolic decay, and dicrotic notch, can be preserved while phase distortion is avoided. Reconstruction from filtered harmonics further suggests that low-order harmonics retain much of the observable waveform structure in the signals analyzed. Overall, this harmonic-based Gaussian filtering provides a promising framework for analysis of PPG signals and motivates further investigation of its potential for extracting physiologically related information from harmonic components, such as bedside monitoring.

## 1. Introduction

Photoplethysmography is a non-invasive optical method used to monitor blood volume changes within the microvascular tissue. It works by illuminating the skin with infrared, red, or green light while a photosensor measures fluctuations in the intensity of light that is either transmitted through or reflected from the tissue. These fluctuations are directly linked to variations in blood flow during the cardiac cycle [1]. Blood volume, pressure, oxygenation levels, and the mechanical properties of the microvascular tissue influence variations in light absorption [2]. The resulting PPG waveform delineates three key events—systole, diastole, and the dicrotic notch—the latter corresponding to aortic valve closure at the end of systole [3].

PPG provides valuable cardiovascular information, including heart rate, oxygen saturation, arterial stiffness, and vascular compliance [4,5]. Owing to its simplicity and low cost, it is widely used in continuous monitoring, wearable devices, and diagnostic applications. The waveform characteristics of the PPG are highly sensitive to physiological and measurement factors, such as the measurement site [6], light wavelength [7], and subject age [8]. It consists of two components: (a) baseline (DC) that is determined by static tissue properties (skin pigmentation, thickness, bone, muscle) and (b) pulsatile (AC) that is caused by blood volume changes with each heartbeat [9].

The pulsatile component of a PPG signal typically constitutes only 1–5% of its total amplitude, making it highly vulnerable to noise from motion artifacts, ambient light fluctuations, and electronic interference [10]. Such noise degrades the SNR, compromising the accuracy of physiological parameter estimation [11,12]. Therefore, effective filtering is essential to isolate a clean and clinically useful pulsatile signal.

The raw PPG signal, including noise, can be decomposed into a sum of sinusoids through Fourier analysis, each characterized by a specific frequency, amplitude, and phase (timing offset). Preserving waveform morphology and precise timing is essential for clinical applications and wearable devices [13]. In particular, the clinical value of PPG signals critically depends on the accurate timing of key pulse features. Events such as the systolic upstroke, dicrotic notch, and diastolic decay are tightly coupled to underlying physiological mechanisms—ventricular ejection, aortic valve closure, and arterial compliance—and occur at well-defined moments within the cardiac cycle [14].

Analogous to the importance of accurate QT interval in electrocardiography (ECG) for assessing repolarization dynamics, the temporal fidelity of PPG features underpins essential metrics such as pulse arrival time (PAT) [8] and pulse transit time (PTT) [8,15], which are widely used for noninvasive blood pressure estimation and arterial stiffness assessment [16,17]. Precision in timing is also required to measure intracranial pressure (ICP) and cerebral blood flow velocity (CBFV) from PPG signals [18,19,20]. Furthermore, beat-to-beat timing precision is crucial for heart rate variability (HRV) analysis [9,16] and arrhythmia detection [4]. When synchronized with ECG, temporally faithful PPG signals enable robust multimodal hemodynamic assessments [3]. Consequently, any denoising or signal enhancement strategy must be carefully designed to preserve the waveform’s timing characteristics. Ensuring temporal fidelity enables the reliable extraction of clinically meaningful cardiovascular insights without compromising the physiological relevance of the signal [21].

Given the critical importance of preserving temporal fidelity in PPG signals, it becomes essential to examine how filtering operations can alter the timing of physiological features. One of the primary sources of timing distortion is nonlinear phase shifting [22]. Phase distortion occurs when a filter introduces non-uniform delays across different frequency components, causing the constituent sinusoids to arrive at different times and ultimately distorting the waveform morphology. In contrast, a filter with linear phase maintains a constant group delay across all frequencies, ensuring that the relative timing of each frequency component remains intact [23]. This property is vital in applications such as audio processing, radar, and biomedical signal analysis, where preserving timing fidelity is essential.

Noise reduction in PPG is most often performed using infinite impulse response (IIR) filters such as Butterworth, Chebyshev, or elliptic designs. While effective in attenuating noise, causal implementations of IIR filters have non-linear phase responses that distort waveform morphology and shift event timing [1,6,24]. Even Bessel filters, among IIR types, have one of the best linear phase responses, but still not perfectly linear [23]. Forward-backward implementation of IIR filters reduces phase delay. However, this renders them non-causal, aggravates edge artifacts at signal boundaries, and the filter response needs to be redesigned to compensate for the double-pass application [25]. Finite impulse response (FIR) filters, in contrast, can maintain linear phase and preserve timing, but generally require high filter orders, which increases the computational load, and are often less effective for noise suppression in short signals [26].

These limitations have motivated alternative strategies, including time–frequency domain processing and nonlinear approaches [27,28]. However, clinical adoption remains limited, in part due to concerns over computational cost, lack of transparency in phase preservation, and loss of physiological detail [22,27].

In this work, we propose a frequency-domain Gaussian filtering framework for harmonic-selective reconstruction of PPG signals. The method suppresses noise while preserving waveform morphology and relative timing of key features. By combining phase-preserving filtering with harmonic-band reconstruction, our framework enables individual PPG harmonics to be isolated and analyzed. As a proof of concept, we evaluate performance using synthetic and experimental PPG recordings, comparing results with conventional IIR filters.

The main contribution extends beyond timing preservation. Specifically, we introduce a harmonic-selective Gaussian filtering framework that allows extraction, selection, and reconstruction of targeted harmonic bands while preserving phase relationships among components. Timing preservation is an inherent property, whereas the broader advance lies in enabling flexible selection of PPG harmonic content.

The remainder of this manuscript is organized as follows. Section 2.1 reviews commonly used FIR and IIR filters for PPG processing and highlights their limitations with respect to phase preservation and waveform fidelity. Section 2.2 presents the properties of Gaussian filters in the Fourier domain and describes the proposed harmonic-based filtering framework. Section 2.3 introduces the empirical model used to simulate PPG signals. Section 3 reports our results for both simulated and experimental PPG data, including comparisons with conventional IIR filtering and an analysis of the physiological information associated with the extracted harmonic components. Section 4 presents our discussion of the advantages and limitations of the proposed method. Finally, Section 5 presents our conclusions.

## 2. Theoretical Background

### 2.1. Filters Commonly Used in PPG Processing

Filtering is fundamental in biomedical signal processing—particularly in photoplethysmography—to suppress noise while preserving physiologically relevant information. Conventional time-domain FIR and IIR filters remain widely used due to their effectiveness in attenuating artifacts. However, their intrinsic limitations, especially regarding phase preservation and waveform fidelity, have motivated the exploration of alternative filtering strategies.

IIR filters are widely used in biomedical signal processing due to their computational efficiency and strong frequency selectivity. Their recursive structure allows a compact implementation of complex transfer functions using fewer coefficients than FIR filters. However, this same feedback mechanism introduces a nonlinear phase response that produces unequal delays across frequency components, and this nonlinearity increases with filter order [29,30,31]. Consequently, timing distortions may arise, and IIR filters could be suboptimal in applications where precise timing preservation is critical.

Among IIR designs, Butterworth filters (orders 2–20) are the most frequently employed due to their smooth passband response [32] and simplicity of implementation [12,33,34,35,36]. However, their moderate roll-off may limit performance in scenarios requiring precise separation of cardiac and motion-related frequency components. Chebyshev type I and II filters (orders 4–20) and elliptic filters (orders 2–20) address this limitation by providing sharper transition bands [1,13,33,37,38,39]. In photoplethysmography, the filters considered most suitable for processing PPG signals are fourth-order Chebyshev type II filters, and Butterworth and elliptic filters, both of which are second-order filters [22].

FIR filters overcome this issue by eliminating feedback, guaranteeing unconditional stability and a linear phase response. In PPG processing, FIR designs with orders up to 64 have been implemented to improve noise attenuation while preserving temporal fidelity [26,40,41]. However, the large number of coefficients increases computational cost and processing latency, limiting their suitability for real-time or resource-constrained applications. In addition, window-based FIR designs (e.g., Hanning, Hamming, Blackman, Kaiser) exhibit ringing artifacts caused by the Gibbs phenomenon, a consequence of approximating sharp spectral transitions with a finite Fourier representation [30]. These artifacts distort the waveform morphology of PPG signals.

Consequently, the trade-off between timing distortion, waveform fidelity, and computational efficiency remains a major limitation of conventional filtering strategies in clinical PPG applications. Although FIR and IIR filters can be designed to target narrow or multiple bands, harmonic-specific selection is typically less direct and may require higher design complexity than the proposed frequency-domain approach. This has two important implications. First, it prevents targeted analysis of the physiological information carried by each harmonic component. Second, the smooth transition between passband and stopband forces designers to increase the filter order to improve noise attenuation, but doing so can result in loss of information, timing distortion, and adverse morphological effects. Thus, selecting an appropriate balance between noise suppression and signal preservation becomes an additional challenge.

A practical advantage of the proposed framework is its direct and adaptive parameterization. The fundamental frequency $f_{0}$ is identified, the harmonic centers are directly defined as $\pm kf_{0}$, and the Gaussian bandwidth is linked to $f_{0}$. Thus, the filter bank is governed primarily by a single signal-adaptive parameter, $f_{0}$. This makes the design straightforward and allows the filter centers and bandwidths to update automatically when the dominant cardiac frequency changes. Its computational cost is implementation-dependent. For a direct fixed-window FFT implementation, the dominant operations are the FFT and inverse FFT, with additional operations required for the K harmonic masks. Alternative implementations, such as sliding-window processing or recursive spectral estimation, are possible but are outside the scope of the present proof-of-concept study.

### 2.2. Gaussian Filters in the Fourier Domain

Throughout this study, we have emphasized the importance of preserving the temporal integrity of PPG signals. To address the limitations of traditional time-domain filters, we utilize the properties of Gaussian filtering in the frequency domain, allowing the signal to be processed and subsequently reconstructed in the time domain without altering its morphology or affecting the precise timing of its features.

A periodic and continuous time-domain signal x(t)=x(t+T) can be expressed through its trigonometric Fourier series representation:

(1) $$x\left(t\right)=a_{0}+\sum_{n=1}^{\infty }\left[a_{n}\cos\left(n2\pi f_{0}t\right)+b_{n}\sin\left(n2\pi f_{0}t\right)\right],$$

where the fundamental frequency is the reciprocal of the period, $f_{0}=1/T$, and the corresponding Fourier coefficients $a_{0}$, $a_{n}$, $b_{n}$ that describe the contribution of each harmonic component are given by Equation (2).

(2) $$\begin{array}{c} a_{0}=\left(\frac{2}{T}\right)\int_{t_{0}}^{t_{0}+T}x\left(t\right)dt,\;\\ a_{n}=\left(2/T\right)\int_{t_{0}}^{t_{0}+T}x\left(t\right)\cos\left(\omega_{n}t\right)dt,\;\\ b_{n}=\left(2/T\right)\int_{t_{0}}^{t_{0}+T}x\left(t\right)\sin\left(\omega_{n}t\right)dt, \end{array}$$

where $\omega_{n}=n2\pi /T$. By reformulating Equation (1) into its complex representation and allowing the period T to approach infinity, the Fourier Transform is obtained, which decomposes the signal into its sinusoidal components:

(3) $$X\left(j\omega \right)=\int_{-\infty }^{\infty }x\left(t\right)e^{-j\omega t}dt,$$

where $j=\sqrt{-1}$ and ω=2πf. In the Fourier domain, the PPG signal S(f), is represented as a set of harmonic components located at integer multiples of the fundamental frequency $f_{0}$, which is determined from the dominant spectral peak within the physiological heart-rate range. The amplitude of each harmonic reflects its contribution to the overall waveform morphology. While it is hypothesized that the first harmonics may contain the most physiologically relevant information, higher-order harmonics were also examined to evaluate this assumption.

Based on this Fourier-domain representation, we define a set of Gaussian filters centered at each harmonic frequency $kf_{0}$, and with Gaussian bandwidth σ. See Equation (4). Each Gaussian is symmetrically applied to the positive and negative frequency components to ensure preservation of phase information during reconstruction. To isolate multiple harmonic bands, individual Gaussian filters are summed, forming a composite frequency-domain filter that selectively retains harmonics of interest. They are expressed by Equation (5).

(4) $$G\left(f\right)=\exp\left[-\frac{{\left(f-f_{0}\right)}^{2}}{\sigma^{2}}\right]+\exp\left[-\frac{{\left(f+f_{0}\right)}^{2}}{\sigma^{2}}\right]$$

(5) $$H\left(f\right)=\sum_{k=-N}^{N}\exp\left[-\frac{{\left(f+kf_{0}\right)}^{2}}{\sigma^{2}}\right],\;k\neq 0$$

where k is the harmonic index, such that k=1 represents the fundamental frequency and subsequent values correspond to its integer multiples. If desired, some bands can be omitted.

In this work, the Gaussian bandwidth is defined relative to the fundamental frequency as $\sigma =\alpha f_{0}$, where α is a dimensionless bandwidth factor. This formulation makes the filter bank adaptive and scale-invariant: when $f_{0}$ changes, both the harmonic centers $k\cdot f_{0}$ and their corresponding Gaussian bandwidths are updated automatically. This is useful for PPG signals, as its spectra are quasi-periodic and exhibit broadened harmonic bands rather than discrete spectral lines, mainly due to physiological variations (including heart-rate variability). A smaller α provides sharper harmonic isolation, whereas a larger α provides smoother reconstruction and greater tolerance to harmonic broadening. In the present proof-of-concept implementation, α = 1/2 was used, corresponding to $\sigma =f_{0}/2$, as a practical compromise between retaining harmonic-band energy and limiting non-harmonic components. Therefore, the value $f_{0}/2$ should not be interpreted as a universal optimum, but as one implementation of the more general adaptive design $\sigma =\alpha f_{0}$.

The DC component at 0 Hz can dominate the spectral magnitude, masking the harmonic structure in the visualization. To address this, we require a high-pass (or low-cut) filter that suppresses the low-frequency contribution while preserving neighboring frequencies. Such a filter can be readily implemented as a Gaussian band-reject filter centered at 0 Hz:

(6) $$\xi \left(f\right)=1-\exp\left[-({f-0)}^{2}/\sigma^{2}\right].$$

Here, $\sigma =f_{0}/2$ remains a practical default value. In general, considering that the cutoff frequency occurs at −3 dB, and the Gaussian definition used here: $\exp\left[-{\left(f-f_{0}\right)}^{2}/\sigma^{2}\right]=1/\sqrt{2}$. Solving for f and substituting the general adaptive relation $\sigma =\alpha f_{0}$, we have $f_{low,high}=\pm \alpha f_{0}\;\surd (\ln\left(\surd 2\right).$ This way, the bandwidth can be obtained directly by selecting α according to the desired spectral broadening, noise level, and harmonic selectivity.

After removing low frequencies, harmonic components become clearly distinguishable, enabling systematic selection of the bandwidths to be analyzed.

Once the desired harmonics have been specified, the filtered spectrum $S_{filtered}\left(f\right)$ is obtained by multiplying the composite Gaussian filter by S(f), which represents the Fourier transform of the raw PPG signal (Equation (7)). The corresponding time-domain signal $s_{filtered}(t)$ is then recovered via the inverse Fourier transform (Equation (8)), yielding a PPG waveform that preserves temporal alignment while attenuating undesired spectral components.

(7) $$S_{filtered}\left(f\right)=S\left(f\right)\cdot H\left(f\right)$$

(8) $$s_{filtered}(t)=F^{-1}\left\{S_{filtered}\left(f\right)\right\}$$

The zero-phase behavior follows directly from the construction of the filter. For a real-valued PPG signal, the Fourier spectrum satisfies $S\left(-f\right)=S^{*}(f)$. The proposed Gaussian mask is real-valued, non-negative, and symmetric, such that H(−f)=H(f). After filtering, conjugate symmetry is preserved and the inverse Fourier transform remains real-valued. Moreover, because H(f) does not introduce a frequency-dependent phase term, the phase of the retained harmonic components is preserved. Therefore, the proposed mask does not impose phase lag or group delay.

This zero-phase behavior holds for the ideal continuous and symmetrically centered Gaussian function [42]. In practical discrete implementations, minor deviations may arise due to sampling resolution, finite-length effects, or numerical approximations rather than from the intrinsic properties of the Gaussian filtering approach [42].

The Gaussian filtering approach exhibits several advantageous mathematical and signal-processing properties. Its exponential profile ensures smoothness by eliminating abrupt spectral transitions and avoiding sharp frequency cutoffs. Unlike rectangular or sharply truncated filters, the continuous decay of the Gaussian function reduces oscillatory artifacts in the reconstructed time-domain signal, thereby mitigating the Gibbs phenomenon. In addition, Gaussian functions provide optimal joint localization in time and frequency, enabling selective harmonic isolation while minimizing spectral leakage. Although the filters are symmetrically centered at harmonic frequencies, the method demonstrates tolerance to tuning variations. Precise tuning is more critical for the fundamental harmonic, whereas higher-order harmonics allow greater flexibility without substantially affecting the reconstructed waveform.

### 2.3. Mathematical Model of the PPG Waveform

Our approach is based on processing the PPG signal in the Fourier domain, where its pulsatile content can be explicitly represented in terms of harmonic components. Previous studies have reported that the physiologically relevant spectral content of the PPG signal is primarily concentrated within the 0.5–10 Hz range, which typically includes the fundamental cardiac frequency and its lower-order harmonics [34,35]. Consequently, the harmonic components collectively shape the overall morphology of the PPG signal, and alterations in their relative amplitudes or phases may affect waveform structure. This harmonic structure motivates the formulation of a compact empirical model that represents the principal pulsatile components of the PPG signal while accounting for realistic acquisition conditions.

In this work, we focus on the dominant low-order harmonics to preserve the principal morphological features of the waveform while minimizing high-frequency noise contributions.

The baseline DC component represents tissue-specific and optical properties, including skin pigmentation, tissue thickness, and contributions from bone and muscle. Superimposed on this baseline, the signal is modulated by a low-frequency respiratory envelope Br(t) representing respiratory-induced variations in blood volume and venous return.

The pulsatile (AC) component, associated with cardiac-driven changes in blood volume, is modeled as the sum of the harmonic components. The fundamental harmonic is defined by an amplitude $a_{1}$, a phase $\phi_{1}$, and a fundamental frequency $f_{0}(t)$, corresponding to the cardiac cycle. Higher-order harmonics occur at integer multiples of this frequency ($2f_{0}(t)$,$3f_{0}(t)$), with corresponding amplitudes $a_{2},a_{3}$ and phase offsets $\phi_{2},\phi_{3}$. Finally, an additive noise term η(t) accounts for motion artifacts and measurement-related disturbances.

The complete empirical model is expressed in Equation (9).

(9) $$PPG(t)=DC+Br(t)+\sum_{m=1}^{N}a_{m}\cos\left[2\pi t\cdot mf_{0}(t)+\phi_{m}\right]+\eta (t)\;$$

where m is the harmonic index (m=1,2…,N), with m=1 corresponding to the fundamental cardiac frequency, and subsequent values correspond to its integer multiples. The fundamental frequency $f_{0}(t)$ is modeled as a time-varying function to account for physiological heart rate variability, allowing the harmonic structure to adapt dynamically to changes in cardiac rhythm.

## 3. Results

As proof of concept, our Fourier-domain framework was first evaluated using a simulated PPG signal and subsequently validated on experimentally acquired recordings. Performance was assessed by comparing the SNR, temporal alignment evaluation, and cross-correlation analysis against conventional IIR filters, including Butterworth, Chebyshev, and elliptic implementations. Signal reconstruction was also compared with forward-backward implementations for zero-phase IIR filtering. All numerical simulations and signal processing were performed using MATLAB R2024.

### 3.1. Synthetic PPG

**Figure 1:**
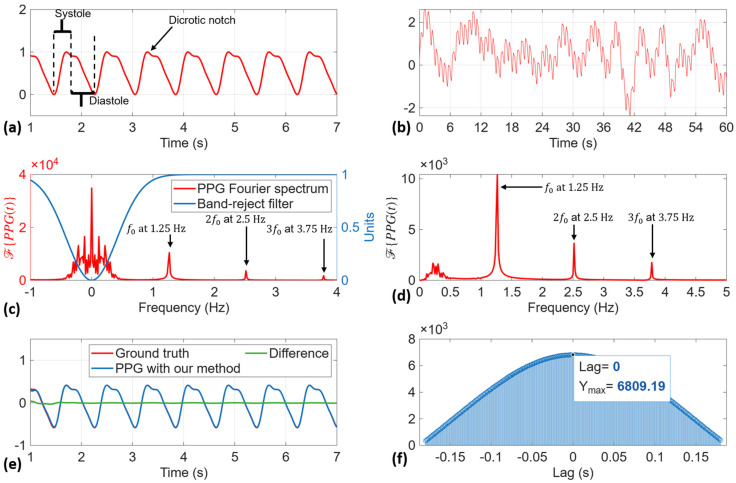
Generation and processing of a synthetic PPG signal. (**a**) Synthetic PPG waveform constructed from three harmonic components of the analytical model. (**b**) Contaminated signal including DC offset, white noise, and respiration-related modulation. (**c**) Frequency-domain representation illustrating DC dominance prior to filtering and its suppression using the proposed band-reject filter. (**d**) Frequency spectrum after DC removal, revealing the fundamental harmonic and subsequent harmonic components. (**e**) Comparison of signal reconstruction using our proposed method and ground-truth signal, illustrating the reconstruction fidelity. (**f**) Cross-correlation between the ground-truth and reconstructed PPG signals, showing maximum correlation at zero lag, thus confirming preservation of temporal alignment.

The synthetic waveform was generated using the analytical model described in Equation (9). Figure 1a shows the clean PPG signal (without added noise), which was subsequently contaminated with white noise, respiration-related modulation, and a dominant DC offset to emulate realistic physiological conditions (Figure 1b). The magnitude of the Fourier spectrum was then computed; however, the harmonic structure was largely masked by the dominant DC component, rendering the harmonic amplitudes barely perceptible. To address this, the DC contribution was removed using the band-reject filter defined in Equation (6), see Figure 1c, enabling clear visualization of the harmonic components in the resulting spectrum (Figure 1d).

The contaminated signal was then processed using the proposed Gaussian-based method. To evaluate the reconstruction fidelity, we computed the point-by-point waveform difference between the ground-truth and reconstructed signal (Figure 1e). The root-mean-square (RMS) of this difference was found to be 6.0 ms, which indicates minimal reconstruction distortion. To assess the temporal alignment, a cross-correlation analysis was performed using MATLAB’s xcorr function (Figure 1f). The maximum correlation occurred at zero lag, confirming temporal alignment between both signals. We also evaluated the alignment of the systolic and diastolic peaks for each detected heartbeat. The timing error was calculated as the absolute difference between the position in the filtered signal and the corresponding position in the ground truth. The calculated root mean square error (RMSE) was 2.84 ms for systolic peaks and 7.67 ms for diastolic peaks.

**Figure 2:**
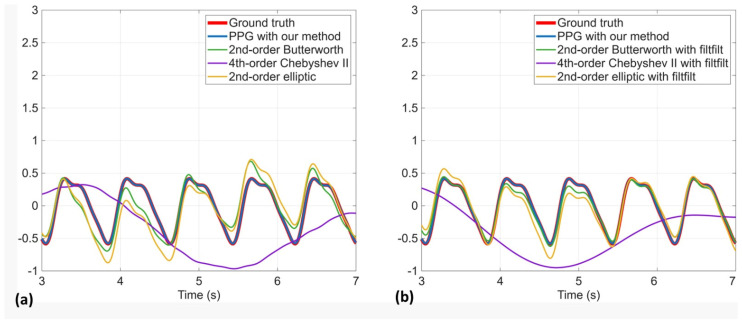
(**a**) Comparison of signal reconstruction using our proposed method and conventional IIR filters, illustrating differences in waveform morphology and temporal alignment. (**b**) Comparison of signal reconstruction using the proposed method and forward-backward implementation of IIR filters. Although temporal alignment is preserved, waveform morphology remains distorted after IIR filtering.

For comparison, conventional IIR bandpass filters were applied in the time domain (Figure 2a). The IIR implementations included a second-order Butterworth and elliptic filter, and a fourth-order Chebyshev type II, all with a bandwidth of 0.6–10 Hz. The IIR filters introduced noticeable morphological deformation and timing shifts, particularly affecting systolic peak position and dicrotic notch visibility. Signal reconstruction was further compared using the same IIR filters implemented in their zero-phase configuration through the filtfilt command, which applies the filter in the forward and reverse directions, thereby canceling phase shifts introduced during filtering. Although temporal alignment was preserved under zero-phase filtering, waveform morphology remained distorted after IIR processing. In contrast, our proposed method showed no apparent temporal displacement or waveform morphology distortion.

Gaussian filtering consistently achieved higher SNR values than the IIR implementations, indicating more effective noise suppression while preserving physiologically relevant temporal characteristics. SNR values were computed using the standard snr function in MATLAB R2024 for all signals analyzed. For this synthetic PPG signal, the SNR increased from 2.13 dB to 50.36 dB after applying the proposed method. By comparison, SNR values obtained using Butterworth, Chebyshev, and Elliptic filters were 2.66 dB, 4.68 dB, and −1.69 dB, respectively. As shown in Figure 2, however, Chebyshev type II failed to preserve the waveform morphology.

These results motivated a more detailed evaluation of noise rejection and temporal fidelity across experimental datasets. The following section examines the performance of Gaussian filtering on real PPG recordings, analyzing its ability to enhance waveform clarity while preserving the timing of clinically relevant pulse features.

### 3.2. Reconstruction Strategy and Preliminary Phase Assessment

To validate the proposed approach on real physiological data, a PPG waveform was acquired and processed using the described Fourier-domain framework. The analysis examined the spectral contribution of individual harmonic components and evaluated filtering performance in terms of SNR and phase preservation.

Signal reconstruction was performed using the first N harmonic bands, where N∈{3,4,…,9,10}, to assess whether higher-order components provided additional morphological detail. A comparison between subsequent reconstructions showed no appreciable differences when preserving harmonic bands beyond the fifth order. Therefore, since a low number of harmonic bands is preferable to improve the SNR, subsequent analyses were conducted using N∈{3,4,5}.

Phase preservation was evaluated by directly comparing the reconstructed waveform with the ground truth PPG signal in the time domain. Visual comparison of the reconstructed and original waveforms showed no obvious displacement of major features such as the systolic peak and dicrotic notch. This behavior is consistent with the symmetric frequency-domain formulation of the proposed Gaussian filtering approach.

### 3.3. Data Acquisition Setup

**Figure 3:**
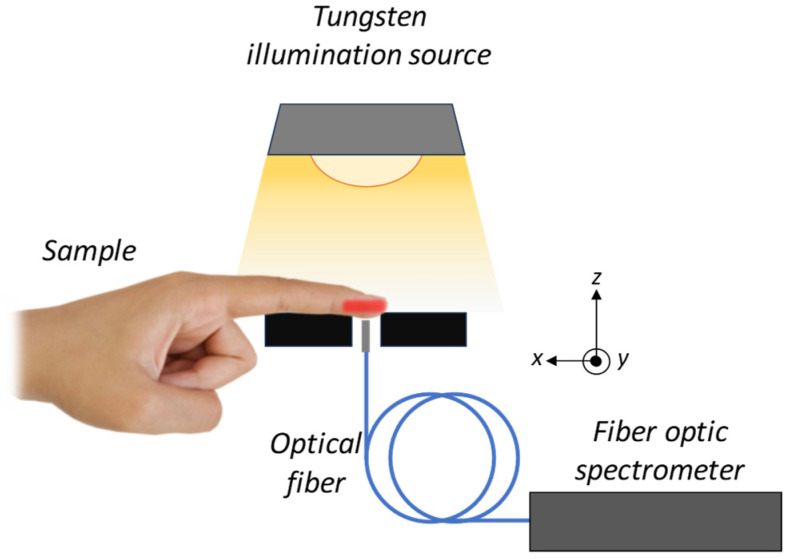
Schematic of the experimental PPG signal acquisition system, comprising: (1) a tungsten light source for fingertip illumination, (2) an optical fiber for light collection and transmission, and (3) a fiber-optic spectrometer for spectral detection.

For proof-of-concept validation of the proposed method, PPG recordings were acquired from three independent participants using a transillumination configuration under typical experimental conditions, inherently subject to physiological variability and measurement noise. Each signal was recorded continuously for 30 s at a sampling frequency of 43.6 Hz. A tungsten light source illuminated the dorsal surface of the fingertip, and the transmitted light was collected through an optical fiber coupled to a fiber-optic spectrometer covering the 200–1000 nm spectral range. For the analysis presented in this study, only the 600–1000 nm wavelength band was considered. The experimental setup is illustrated in Figure 3. Detailed reconstruction results are presented for one representative recording to illustrate the full processing pipeline. The remaining acquisitions are included in the following subsection to demonstrate consistency in harmonic extraction and waveform preservation across different subjects. During acquisition, heart rates were approximately 80 bpm, with minor inter-subject variation.

### 3.4. Noise Reduction, Timing Preservation, and Waveform Fidelity

**Figure 4:**
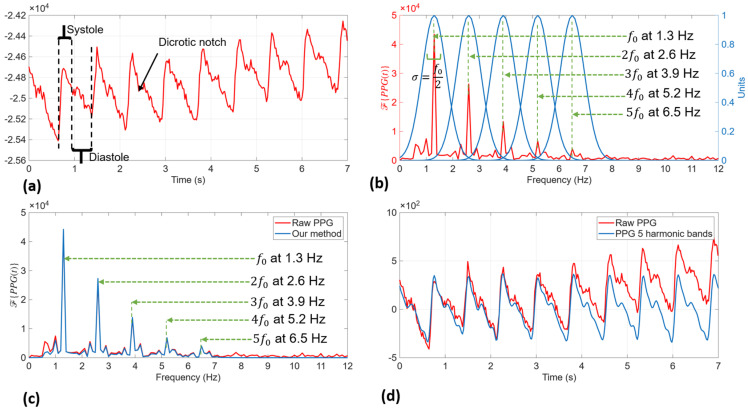
(**a**) Raw transillumination-acquired PPG signal exhibiting substantial noise that obscures key morphological features like the dicrotic notch. (**b**) Fourier transform of the PPG signal, clearly illustrating the harmonic components (red line), which facilitates the accurate extraction of signal information separated from the rest of the noise. The Gaussian profile for the first five harmonics (blue line) is shown. (**c**) Magnitude spectrum of the raw PPG (red) and with the first five harmonics extracted (blue). Note that frequencies below 0.5 and above 5f0 were eliminated, while extracted harmonic components retain their intrinsic spectral shape following Gaussian filtering. (**d**) PPG signal obtained after applying the proposed method (blue line) compared with the raw PPG signal (red line). A vertical offset was applied to align the raw signal with the signal reconstructed using our method.

The transillumination-acquired PPG signal exhibited substantial noise, which masked accurate identification of the dicrotic notch (Figure 4a). Because effective filtering must suppress noise without altering waveform morphology or temporal characteristics, the signal was analyzed in the Fourier domain to separate physiologically meaningful harmonic components from broadband noise.

The clear visualization of the harmonics of the PPG signal facilitates their extraction using Gaussian filters, enabling separation of physiologically meaningful components from noise. Gaussian filters were centered at the fundamental frequency and its subsequent harmonics to isolate the first five harmonic bands while attenuating out-of-band spectral content (Figure 4b), according to Equation (5). The smooth spectral transitions of Gaussian filters preserve the intrinsic shape of each extracted harmonic in the Fourier domain, as illustrated in Figure 4c.

The reconstructed signal was then obtained by applying the Inverse Fourier Transform to the filtered harmonic components, as described in Equation (8). This reconstruction demonstrated a marked enhancement in waveform clarity. In particular, the dicrotic notch became clearly distinguishable (Figure 4d), consistent with observations from the synthetic signal analysis. Temporal fidelity was evaluated by directly comparing the reconstructed waveform with the ground truth PPG signal in the time domain. The alignment of characteristic features, including the systolic peak and dicrotic notch, indicated the absence of observable temporal displacement, confirming that the proposed symmetric Gaussian filtering preserves timing information. The SNR increased from 10.37 dB in the original signal to 22.30 dB after harmonic isolation. This large increase shows the effective suppression of out-of-band spectral components under controlled harmonic reconstruction, rather than a physiologically representative absolute SNR value.

**Figure 5:**
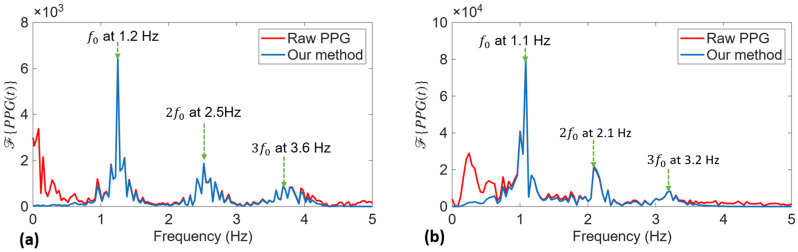
(**a**) Magnitude spectrum of another representative PPG signal. The raw PPG is represented by the red line, while the first three harmonics extracted are represented by the blue line. In this case, the fourth- and fifth-order harmonics exhibit considerably smaller amplitudes, so we decided to preserve just the first three harmonic bands. (**b**) Magnitude spectrum of a representative PPG signal, which was recorded from a different subject. Just as in the above case, we extracted the first three harmonics. The red line shows the magnitude spectrum of the raw PPG, and the blue line shows the first three harmonics extracted.

Figure 5a,b show other representative PPG signals recorded from different subjects to demonstrate the preservation of the intrinsic shape of each extracted harmonic in the Fourier domain. In these cases, the fourth- and fifth-order harmonic bands exhibit considerably smaller amplitudes, so only the first three harmonic components were used for the signal reconstruction. Additionally, the slow baseline drift present in the original recordings was effectively suppressed after Gaussian filtering.

Preservation of harmonic shape in the frequency domain ensures retention of the physiological information encoded in each component, which explains why waveform morphology remains stable after signal reconstruction.

**Figure 6:**
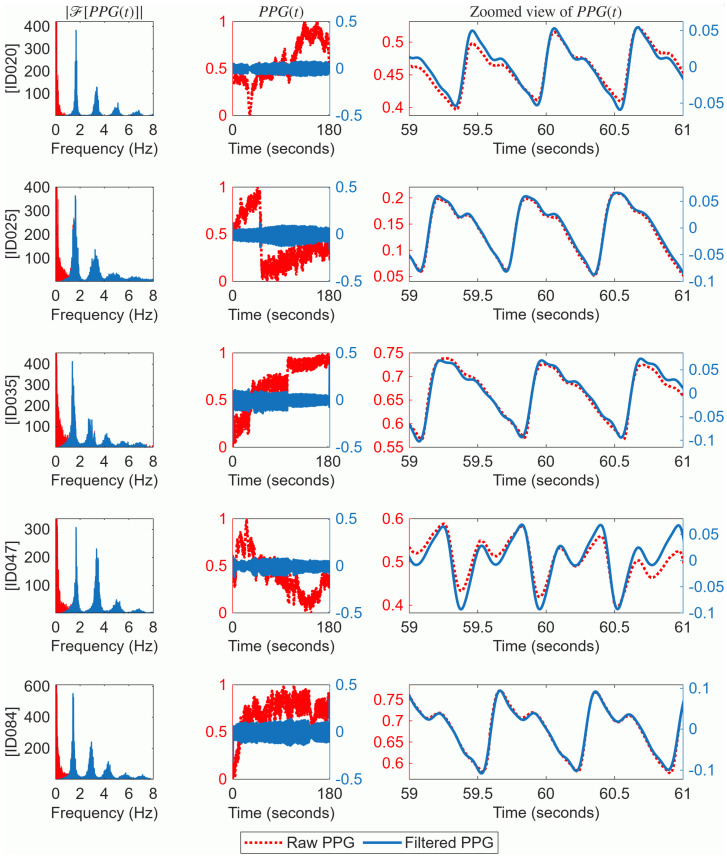
Fourier spectra, 180-s PPG recordings, and zoomed-in views around the 60-s mark, (raw in red, filtered in blue) for five test subjects taken from a public database [44]. Here, our Gaussian filtering selectively preserves the first five harmonic bands.

To further validate our method experimentally, we also analyzed PPG recordings extracted from a database published on Mendeley Data by Shiyong Li [43]. This database includes 148 test subjects, with 180-s PPG recordings taken at 250 Hz. Due to space constraints within this manuscript, we have selected five representative test subjects, whose anonymized data are presented in Table 1. The data include a three-digit identifier, age, sex, height (in centimeters), weight (in kilograms), systolic and diastolic blood pressure (in millimeters of mercury), and average heart rate (in beats per minute). Without loss of generality, we selected PPG recordings taken after physical exercise and captured using the infrared channel [43]. The raw PPG recordings and the result of applying our selective harmonic-band Gaussian filtering are shown in Figure 6.

The first column of Figure 6 presents the spectra of the raw and filtered PPG recordings. The vertical scale was adjusted identically in both plots to visualize the harmonic bands in detail. For this reason, the baseline peak at 0 Hz is off the scale (its maximum was up-to 50 times as large as that of the first harmonic). The simultaneous plots on the second column show that the raw data exhibit considerable baseline drift, as well as artifacts related to movement and respiration. In contrast, our filtered data remain quasi-stationary. The second and third columns of this figure show that the proposed filtering preserves the variability of the wavefront morphology.

### 3.5. Application-Driven Selection of the Number of Harmonic Bands

Selecting the number of harmonic bands N is important because it directly determines the trade-off between reconstruction detail and spectral complexity. Because the PPG signal is quasi-periodic, its spectrum is more appropriately described as broadened harmonic bands centered near integer multiples of the cardiac fundamental $f_{0}$, rather than ideal discrete spectral lines; accordingly, the number of retained bands depends on the physiological endpoint of interest and the available computational budget [1]. Within the proposed framework, this choice is implemented by constructing symmetric Gaussian masks centered on the desired harmonic bands (and their negative-frequency counterparts), which enables the selective retention of low-order components while preserving temporal alignment under Fourier-domain implementation. When the endpoint is limited to heart rate tracking (e.g., dominant frequency or phase-derived instantaneous rate), retaining only the fundamental band may be sufficient in principle.

**Figure 7:**
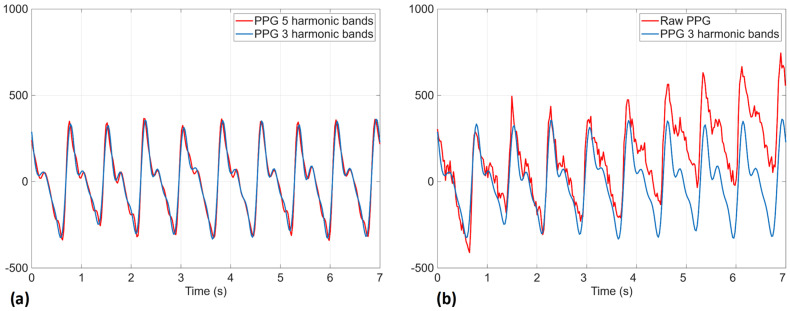
(**a**) Comparison between PPG signals reconstructed from five (red line) and from three harmonic bands (blue line), showing similar waveform morphology while temporal alignment is maintained. (**b**) PPG signal reconstructed using the first three harmonics, demonstrating that the principal waveform morphology and temporal alignment (phase) are preserved, with enhanced waveform clarity and improved visibility of the dicrotic notch.

An illustrative example is shown in Figure 7a, where reconstructions using three and five harmonic bands remain time-aligned and exhibit similar morphology; this comparison is intended to visualize the practical effect of varying N, not to assert completeness of any specific harmonic order. Prior works provide guidance on the choice of N. An integrative review [1] reports that most PPG waveform energy is contained within the first three harmonics. In line with common preprocessing practice, Gao (2025) summarizes high-frequency filtering choices that set the upper cutoff near 10 Hz [44], corresponding approximately to the fourth harmonic at 150 bpm or the third harmonic at 200 bpm (i.e., N ≈ 3–4 as a practical setting).

Conversely, applications that analyze harmonic indices may require retention of higher-order components (e.g., up to the 12th harmonic), even though reported harmonic ratios decay rapidly beyond the third or fourth harmonic (with the ratio of the fourth-harmonic amplitude to the first (HA_4_/HA_1_) on the order of 10^−3^, and higher orders approaching 10^−5^ or smaller), indicating diminishing returns unless subtle high-order content is itself the endpoint [45]. In practice, N should therefore be selected based on the target application and available computational resources, using fewer bands for efficient morphology-preserving filtering (as in Figure 7b), and more bands when finer waveform detail or harmonic-index features are required.

### 3.6. Robustness of Gaussian Filtering to Frequency Detuning

**Figure 8:**
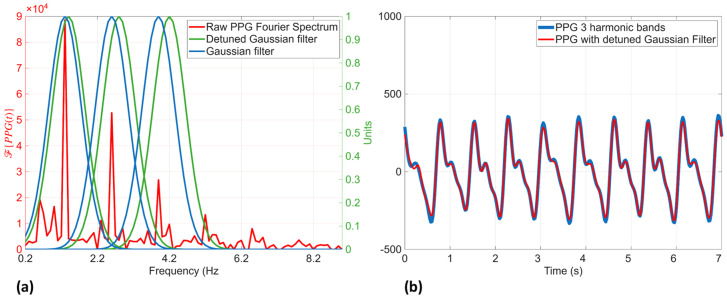
(**a**) Evaluation of filter robustness under detuning conditions, showing that precise tuning is required primarily for the fundamental harmonic, while higher-order harmonics tolerate moderate frequency offsets. (**b**) Reconstructed PPG waveform obtained after detuning the Gaussian filters corresponding to the second and third harmonics, demonstrating preservation of temporal alignment (phase) despite frequency misalignment.

The proposed Gaussian filtering strategy was validated using PPG signals affected by experimental noise and physiological variability. In the preceding analyses, each Gaussian filter was centered on its corresponding harmonic frequency. In practice, the first harmonic peak is generally readily identifiable once the DC component and low-frequency contamination have been suppressed. Nevertheless, to assess the effect of imperfect alignment between the spectral peak and the Gaussian filters, the filter corresponding to the fundamental harmonic was intentionally detuned by ±0.1 Hz with respect to the detected peak frequency. The filters associated with the second and third harmonics were then detuned proportionally by ±0.2 Hz and ±0.3 Hz, respectively, consistent with their dependence on $f_{0}$ (Figure 8a).

Despite these perturbations, the reconstructed PPG waveform remained visually aligned with the original signal, with no evident timing distortion in the principal pulse features. These results further suggest that higher-order harmonics, which carry less spectral energy and contribute less to the overall temporal structure of the waveform, may tolerate comparable relative frequency offsets without materially compromising temporal fidelity. In particular, the temporal locations of the systolic upstroke, systolic peak, and dicrotic notch appeared to be preserved after reconstruction, indicating that detuning of the Gaussian filters by approximately 10% of the fundamental frequency did not substantially modify the phase relationships among the retained spectral components (Figure 8b).

### 3.7. Exploratory Observations on Harmonic Waveform Contributions

**Figure 9:**
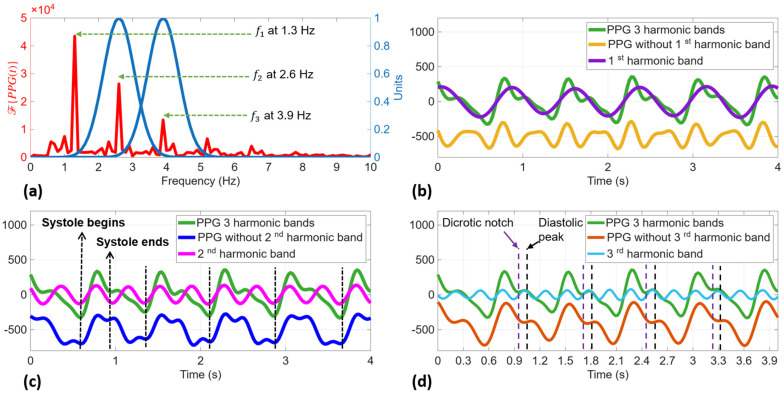
(**a**) Example of selective harmonic extraction using the proposed Fourier-domain Gaussian filtering approach. Gaussian filters (blue line) were positioned on the second and third harmonic bandwidths (red line) to visualize the contribution of the first harmonic to the PPG waveform morphology. (**b**) Extracted first harmonic bandwidth (purple line) shown alongside the full PPG signal (green line) and the PPG signal reconstructed after exclusion of the first harmonic (yellow line). (**c**) Extracted second harmonic bandwidth (magenta line) compared with the full PPG signal (green line) and the PPG signal reconstructed after exclusion of the second harmonic (dark line). The valleys of the second harmonic align with the onset and termination of systole in the PPG waveform. (**d**) Extracted third harmonic bandwidth (blue line) contrasted with the full PPG signal (green line) and the PPG signal reconstructed after removal of the third harmonic (orange line). The peak of the third harmonic aligns with the diastolic peak of the PPG waveform, occurring immediately after the dicrotic notch. Exclusion of the third harmonic affects the temporal accuracy of the notch, which could suggest an association with events related to aortic valve closure. Here, a vertical offset was applied for ease of visualization.

Since the PPG signal can be reconstructed from the first three harmonics, we examined the potential physiological information carried by each individual harmonic. To this end, Gaussian filters were positioned on selected harmonic components to isolate them in the Fourier domain. An example of this procedure is shown in Figure 9a, where the second and third harmonics are selectively extracted, allowing visualization of the contribution of the first harmonic to the overall PPG waveform. This procedure was systematically applied to each of the first three harmonics.

Figure 9b–d presents three signals in each panel: (1) the PPG signal reconstructed from the first three harmonic bandwidths, (2) the PPG signal reconstructed after isolating the selected harmonic components, and (3) the excluded harmonic bandwidth.

Figure 9b suggests that the first harmonic plays a dominant role in shaping the global morphology of the PPG waveform. During data acquisition, the participant’s heart rate was measured at 80 bpm. Mapping this value to the frequency domain yields an equivalent frequency of approximately 1.3 Hz, which coincides with the frequency of the first harmonic observed in the Fourier spectrum (Figure 9a). Then, removal of the first component results in pronounced waveform distortion (yellow line), which may indicate that it carries most of the information associated with the fundamental cardiac rhythm. We plotted the first harmonic of the PPG signal to identify correspondences between points on the first harmonic graph (purple line) and the PPG signal constructed using the first three harmonics (green line). No simple one-to-one correspondence was observed between key points (diastole, systole, and notch) on the PPG signal and the first harmonic graph. This could indicate that there is no association between the first harmonic and these key events in the PPG signal.

Figure 9c could suggest that the valleys of the second harmonic (magenta line) align with the onset of systole in the PPG signal (green line). In addition, the intersections between the second harmonic and the reconstructed PPG waveform correspond to the end of systole. These observations may indicate that the second harmonic contains information related to both the initiation and termination of the systolic phase. As in the previous case, the absence of the second harmonic indicates distortion of the PPG signal (dark blue line).

Figure 9d shows that, in the reconstructed PPG waveform (green line), the third harmonic (blue line) reaches its maximum amplitude at the same temporal location as the diastolic peak, which occurs immediately after the dicrotic notch. Excluding this component introduces a slight temporal shift in the notch region (orange line), suggesting that the third harmonic may contribute to preserving the accurate timing of events associated with aortic valve closure.

Overall, this harmonic extraction analysis provides a framework for interpreting physiological content within individual spectral components of the PPG signal. The first harmonic may correspond to the fundamental cardiac rhythm, the second harmonic appeared to align with systolic timing in this representative example, and the third harmonic appeared to influence waveform shape near the dicrotic-notch region.

## 4. Discussion

The proposed harmonic-selective Gaussian filtering framework enables the extraction, selection, and reconstruction of specific PPG harmonic bands, or combinations of harmonic bands, while preserving the phase relationships among the retained components. Timing preservation is an inherent analytical property of the framework because, by construction, it does not introduce frequency-dependent phase terms. Nevertheless, the reconstructed waveform may still differ because of harmonic selection, finite window length, sampling resolution, spectral leakage, noise, and landmark detection uncertainty.

We emphasized visual inspection because waveform morphology remains clinically relevant in biomedical signal interpretation. In diagnostic or clinical contexts, specialists often rely on the apparent morphology and timing of features such as the systolic peak, diastolic decay, and dicrotic notch. Therefore, visual preservation of these features provides practical waveform interpretability. Having established the importance of morphological fidelity, we next consider how our specific implementation choices affect that fidelity in practice.

In the present implementation, the Gaussian harmonic masks are applied to a fixed-window Fourier-domain representation of the PPG signal. This implementation is appropriate when the analyzed segment can be considered locally quasi-stationary, such as short-term recordings acquired under controlled conditions or offline analysis of selected PPG segments. However, the proposed framework is not intrinsically restricted to fixed-window processing. The same principle can be extended to sliding-window Fourier or short-time Fourier transform implementations. In such cases, $f_{0}$, the harmonic centers $\pm kf_{0}$, and the Gaussian bandwidths may be updated for each window to follow temporal changes in the harmonic structure. Regardless of the windowing strategy, a key advantage of this framework over conventional filter designs is its ability to preserve waveform timing and morphology while reducing distortion.

## 5. Conclusions

In this work, we proposed a Fourier-domain Gaussian filtering framework for selective harmonic extraction of PPG signals. As a proof of concept, the proposed framework was evaluated using both a simulated PPG waveform and experimentally acquired PPG recordings. The method suppresses the dominant DC contribution, reduces drift of the non-pulsatile baseline, and attenuates low-frequency contributions while enabling the isolation of specific harmonic bands with preserved phase relationships among the retained components.

In the synthetic and experimental signals analyzed, our approach improved waveform clarity, enhanced visibility of relevant pulse features, and maintained visual temporal alignment of key features, including the systolic upstroke, systolic peak, and dicrotic notch. The results further indicated that the principal waveform morphology can be satisfactorily recovered from a number of low-order harmonics, suggesting that much of the relevant structural information is concentrated in the lowest spectral components.

A practical advantage of the proposed framework is that it can be matched directly to the center frequency of each harmonic component in a given PPG signal. Unlike traditionally used filters, whose passband is typically fixed after design and often requires coefficient recalculation to modify, Gaussian filters can be centered on the detected harmonic peaks through a single parameter, making them easier to adapt to spectral shifts caused by physiological variability. The detuning analysis further suggested that the framework is tolerant to frequency offsets, including for higher-order harmonics, whose lower spectral energy has a reduced influence on the global temporal structure of the signal.

The proposed framework is grounded in a well-established mathematical basis derived from the Fourier-domain representation of the PPG signal. Results of our proof of concept support the use of harmonic-selective Gaussian filtering as a promising approach for morphology-preserving PPG analysis. Future work should focus on broader experimental validation across larger subject populations, broader acquisition conditions, and quantitative assessment of timing preservation.

## Figures and Tables

**Table 1:** Anonymized information of 5 test subjects extracted from [44].

ID	Age	Gender	Height /cm	Weight /kg	SBP /mmHg	DBP /mmHg	HR /bpm
020	25	Female	158	50	115	68	75
025	25	Female	160	64	114	58	75
035	23	Female	160	52	100	66	77
047	25	Male	185	80	107	74	83
084	23	Male	173	80	112	68	83

## Data Availability

The raw data supporting the conclusions of this article will be made available by the authors on request.
